# Relationship between Dietary Creatine and Growth Indicators in Children and Adolescents Aged 2–19 Years: A Cross-Sectional Study

**DOI:** 10.3390/nu13031027

**Published:** 2021-03-23

**Authors:** Darinka Korovljev, Valdemar Stajer, Sergej M. Ostojic

**Affiliations:** 1 Applied Bioenergetics Lab, Faculty of Sport and Physical Education, University of Novi Sad, 21000 Novi Sad, Serbia; korovljev.darinka@gmail.com (D.K.); stajervaldemar@yahoo.com (V.S.); 2 Faculty of Health Sciences, University of Pecs, H-7621 Pecs, Hungary

**Keywords:** creatine, children, height, BMI-for-age, stature-for-age, growth

## Abstract

A possible role of dietary creatine for ensuring proper growth and development remains unknown. The main aim of this cross-sectional study was to quantify the amount of creatine consumed through regular diet among U.S. children and adolescents aged 2 to 19 years and investigate the relationship between creatine intake and growth indicators, using data from the 2001–2002 National Health and Nutrition Examination Survey (NHANES). We included data for NHANES 2001–2002 respondents (4291 participants, 2133 boys and 2158 girls) aged 2 to 19 years at the time of screening, who provided valid dietary information and examination measures (standing height and weight). Individual values for total grams of creatine consumed per day for each participant were computed using the average amount of creatine (3.88 g/kg) across all sources of meat-based foods. All participants were categorized for height-for-age and BMI-for-age categories. The average daily intake of creatine across the whole sample was 1.07 ± 1.07 g (95% CI, from 1.04 to 1.10). Height, weight, and BMI were significantly different across creatine quartiles (*p* < 0.001), with all measures significantly higher in the 4th quartile of creatine intake (≥1.5 g/day) than those in other quartiles (*p* < 0.05). The participants from the 3rd quartile of creatine intake (0.84–1.49 g/day) were significantly different from others with respect to having lower rates of normal stature and higher rates of tall stature (*p* < 0.05). Each additional 0.1 g of creatine consumed per day increases height by 0.60 cm (simple model) or 0.30 cm (adjusted model). The daily intake of creatine from a regular diet in taller children and adolescents was higher than in shorter peers aged 2–19 years. Future research has to monitor temporal changes in growth and dietary creatine and validate our findings in interventional studies across pediatric populations.

## 1. Introduction

Providing a diet with a variety of food components plays a crucial role in supporting normal growth in children and adolescents [1]. Besides meeting the high energy requirements and supplying essential macro and micronutrients, increasing evidence has shown that humans do have dietary needs of non-essential amino acids and by-products to fulfill their genetic potential for maximum growth, as well as optimal health and well-being [2,3]. Of particular note, adequate provision of dietary creatine may be necessary for optimizing human growth, development, and health. Creatine is a non-proteinogenic amino acid derivative that occurs naturally in the human body. About half of its daily turnover comes from a carnivorous diet while another half is synthesized endogenously in the liver, kidney, and pancreas, maintaining a creatine homeostatic load of ~2 g per day for an average person [4]. Creatine is a pleiotropic nutraceutical that plays an essential role in several metabolic pathways, including tissue bioenergetics and cellular growth. Its contribution to early growth and development is apparent throughout the literature, with de novo and dietary creatine necessary to uphold optimal placental function, maintenance of pregnancy, as well as fetal growth and maturation [5]. Dietary creatine is also proving to effectively tackle creatine deficiency syndrome, a genetic malfunction of creatine biosynthetic enzymatic machinery characterized by developmental delay and neuromuscular manifestations, allowing neonates and youngest children with the condition to thrive [6]. Still, whether dietary creatine affects growth in older children and adolescents at the populational level has been to date mostly unaddressed. A handful of previous small-scale studies demonstrated the efficacy of supplemental creatine among athletic adolescents and pediatric patients [7,8]; however, research in this area omitted to provide any data about dietary creatine intake and growth. Therefore, the purpose of this cross-sectional study was to quantify the amount of creatine consumed through regular diet among U.S. children and adolescents aged 2 to 19 years and investigate the relationship between creatine intake and growth indicators, using data from the 2001–2002 National Health and Nutrition Examination Survey (NHANES).

## 2. Materials and Methods

### 2.1. Study Population

Data for this study were obtained from the NHANES 2001–2002 round. NHANES is an annual survey research program operated through the U.S. National Center for Health Statistics, Division of Health Examination Statistics, a part of the Centers for Disease Control and Prevention (CDC). The program is created in 1959 to assess children and adults’ health and nutritional status in the United States. The principal objective of NHANES is to estimate the number and percent of persons in the U.S. population and designated subgroups, with selected diseases and risk factors, and monitor trends in the prevalence, awareness, treatment, and control of selected conditions. The NHANES 2001–2002 round included 11,039 civilian, non-institutionalized male and female individuals aged 0 to 85 years. For this report, we sorted out data for respondents aged 2 to 19 years at the time of screening, who provided valid dietary information and examination measures (see below). The data collection for NHANES 2001–2002 was carried out between January 2001 and December 2002, with informed consent obtained from all participants or parents/guardians. The ethical approval to conduct the NHANES 2001–2002 was granted by the NHANES Institutional Review Board (Protocol #98-12).

### 2.2. Dietary Data

Dietary intake information was acquired from the NHANES 2001–2002 Dietary Data component. The dietary intake data were used to estimate the types and amounts of foods and beverages consumed during the 24 h before the in-person interview (midnight to midnight) and to calculate intakes of energy, relevant nutrients, and other food components from foods and beverages consumed; proxy interviews were conducted for survey participants less than six years of age, and assisted interviews were conducted with survey participants 6 to 11 years of age. To calculate creatine intake, we first identified meat-based protein foods using 8-digit food codes from the U.S. Department of Agriculture (USDA) using dietary interview entries for individual foods; red meat, poultry, fish, and seafood are recognized as the primary sources of dietary creatine [9,10]. We subsequently recorded the gram weight of each food component containing meat (USDA codes from 20000000 to 28522000) and calculated the net intake of those foods for each individual by merging all relevant food items on a daily basis. Individual values for total grams of creatine consumed per day for each participant were computed using the average amount of creatine (3.88 g/kg) across all meat sources, as previously described [11]. Nutrient intakes reported did not include those obtained from dietary supplements, medications, or plain drinking water. The intakes were calculated using the USDA Food and Nutrient Database for Dietary Studies, which contains the most up-to-date food composition values available for this time frame. The primary exposure used in this report was the mean grams of creatine consumed per day, and the secondary exposure included the average daily dietary intake of creatine categorized into quartiles.

### 2.3. Body Measurements

Data for this domain were acquired from NHANES 2001–2002 Examination Data component for body measures. At a minimum, body weight and standing height were measured for all participants. Body weight was taken in underwear using a digital scale (Mettler Toledo, Columbus, OH, USA), with readings recorded to the nearest 0.1 kg by the automated system. Standing height was measured with a stadiometer in the Frankfort horizontal plane to the nearest 0.1 cm (Seca, Chino, CA, USA). All measures were obtained by trained health technicians using a standardized protocol with calibrated equipment, and measurement components performed in a specially equipped room in the mobile examination center. Detailed NHANES body measurements component procedures are presented elsewhere [12]. Body mass index (BMI) was calculated for each participant as weight in kilograms divided by the square of height in meters (kg/m^2^). For height and BMI, we also calculated individual percentiles for each participant, using CDC growth charts for ages 2–20 years [13], based on CDC LMS tables tabulated at half-month intervals. The participants were further categorized for height-for-age percentiles as short stature (height-for-age less than the 5th percentile), normal stature (5th to less than the 95th percentile), and tall stature (equal to or greater than the 95th percentile). The weight status categories and the corresponding percentiles were as follows: underweight (BMI-for-age less than the 5th percentile), normal weight (5th percentile to less than the 85th percentile), overweight (85th to less than the 95th percentile), and obese (equal to or greater than the 95th percentile). The primary outcome used in the analyses was height; the secondary outcomes include weight, BMI, height-for-age, and BMI-for-age. Height-for-age and BMI-for-age are recognized as common growth indicators in children and adolescents by the World Health Organization [14].

### 2.4. Statistical Analyses

Descriptive statistics were used to describe the characteristics of the study population. Data series were analyzed by Kolmogorov–Smirnov test for normality of distribution. Kruskal–Wallis non-parametric one-way ANOVA was used to compare body measures across creatine quartiles, and dietary creatine intake across different growth categories, with post hoc pairwise comparison tests employed to identify the differences between individual sample pairs. Single and multiple regression analyses with entering procedures were conducted to assess the association between creatine intake and growth indicators. The regression models were adjusted for an a priori defined set of covariates, including demographic variables (gender, race/ethnicity, annual household income), and nutritional variables (energy, total protein). Finally, chi-square cross-tabulation analysis was conducted for comparing observed frequencies for growth indicators (stature-for-age and BMI-for-age categories) across different creatine quartiles, with individual proportions compared with Z-test adjusted for standardized residuals. Data were analyzed using SPSS Statistics for Mac (Version 24.0) (IBM, Armonk, NY, USA), with the significance level set at *p* < 0.05, and all statistical tests were two-sided.

## 3. Results

A total of 4291 participants aged 2 to 19 years (2133 boys (49.7%) and 2158 girls (50.3%)) provided individual dietary data and were assessed for body weight and standing height. Table 1 displays the basic demographic and nutritional characteristics of the study sample. The mean age was approximately 11 years of age, and the mean caloric intake was ~2100 kcal per day. The average daily intake of creatine across the whole sample was 1.07 ± 1.07 g (95% confidence interval, from 1.04 to 1.10). The average creatine intake was categorized into quartiles, ranging from 0.00–0.28 g (1st quartile; mean ± SD = 0.06 ± 0.09 g), 0.28–0.83 g (2nd quartile; 0.56 ± 0.16 g), 0.84–1.49 g (3rd quartile; 1.13 ± 0.19 g), and 1.50–8.28 g (4th quartile; 2.52 ± 1.06 g).

**Figure 1:**
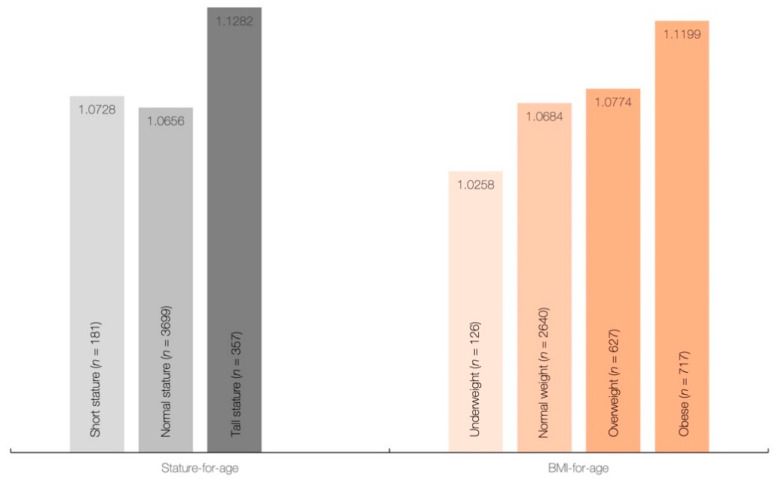
Dietary intake of creatine (g/day) across stature-for-age and BMI-for-age categories.

Height, weight, and BMI were significantly different across creatine quartiles (*p* < 0.001), with differences between individual sample pairs for each body measure depicted in Table 2. All measures were significantly higher in the 4th quartile of creatine intake than those in other quartiles (*p* < 0.05), with differences for other interquartile comparisons displayed a significant downward trend towards the first quartile of creatine intake (Q3 > Q2 > Q1) for most variables (*p* < 0.05). No significant differences were found in mean dietary creatine intake across different stature-for-age categories (*p* = 0.154), and BMI-for-age categories (*p* = 0.138) (Figure 1), although daily creatine consumption tended to be higher in children and adolescents with tall stature and obese individuals.

**Figure 2:**
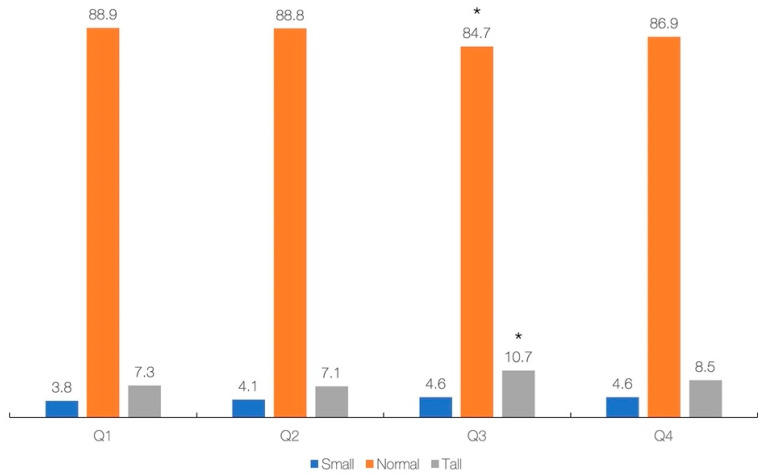
Dietary intake of creatine (g/day) across stature-for-age. Creatine intake across quartiles was 0.00–0.28 g (Q1), 0.28–0.83 g (Q2), 0.84–1.49 g (Q3), and 1.50–8.28 g (Q4). Asterisk (*) indicates a significant difference at *p* < 0.05 between quartiles.

The results of regression analysis with simple and multivariable models (adjusted for gender, ethnicity, annual income, energy intake, and total protein) showed a significant association between primary exposure (dietary creatine intake) and most primary and secondary outcomes across the whole sample (Table 3), except for BMI-for-age in the crude model (*p* = 0.227), and height-for-age in the adjusted model (*p* = 0.460). Each additional 0.1 g of creatine consumed per day increases height by 0.60 cm (simple model) or 0.30 cm (adjusted model). Finally, the cross-tabulation analysis revealed a significant difference across creatine quartiles and stature-for-age categories (*p* = 0.039), with participants from Q3 are significantly different from others with respect to having lower rates of normal stature and higher rates of tall stature (*p* < 0.05) (Figure 2). No significant difference was found across creatine quartiles and BMI-for-age categories (*p* = 0.483).

## 4. Discussion

To our knowledge, this is the first cross-sectional populational study that evaluated the association between daily intake of creatine from regular diet and growth indicators in U.S. children and adolescents aged two years and over. We found that children and adolescents with a higher intake of creatine have higher stature, weight, and BMI compared to lower intake peers, following a stepwise rise corresponding to an incremental increase in dietary creatine intake. The mean dietary creatine intake appeared similar across different stature-for-age and BMI-for-age categories; however, the participants in the 3rd quartile of creatine intake (0.84–1.49 g/day) were significantly different from others with respect to having higher rates of tall stature. After controlling for demographic and nutritional variables, dietary creatine intake appeared positively associated with most growth indicators.

Creatine (methyl guanidino acetic acid) is a nitrogen-containing metabolite of arginine, glycine, and methionine synthesized endogenously in the human body and acquired through diet. Growing evidence suggests that creatine, along with other amino acid derivatives originating from animal-based foods, may play an essential role in human growth and development, from intrauterine growth onwards [10]. A preliminary metabolomics study advances normal maternal creatine levels during the third trimester of pregnancy as a protective factor against poor perinatal outcomes, including a small-for-gestational-age infant, preterm birth, and neonatal intensive care admission [15]. A retrospective cohort study shows that fetal growth is positively associated with creatine levels, with each unit increase in maternal creatine goes with a 1.23 unit increase in birthweight centile and a 0.11-cm increase in birth length [16]. These studies perhaps point to increased requirements for creatine due to the fetus’s rapid growth and increased metabolic needs throughout pregnancy. Another prospective study demonstrated an increase in fetal brain creatine levels in healthy pregnant women between 18- and 40-weeks gestational age who underwent proton-MRS [17]. This continues for the postnatal period, with cerebral creatine increment occurs during the first months of life and growth [18]. Creatine appears to be related to the normal growth of older children as well. In a small-scale trial, 19 undernourished boys aged 8–11 years demonstrated low creatine levels due to diet deficient in creatine-building components, with nutritional supplementation provides compensation for creatine levels towards weight gain [19]. Dietary creatine normalizes creatine concentrations, makes substantial developmental progress, and attenuates clinical features in children suffering from creatine deficiency syndromes [6]. In addition, supplemental creatine elicited a significant increase in height in children with acute lymphoblastic leukemia [20], implying the importance of creatine in the growth and maturation across various pediatric settings.

Our results corroborate previous findings concerning the positive association between dietary creatine and growth in a nationally representative cohort of children and adolescents. The link between food creatine and growth remained robust even after controlling for energy and total protein, suggesting a distinctive role of dietary creatine during growth. We hypothesized that creatine might favorably affect growth by several means that involve augmented energy metabolism [21], bone mass accretion [20], and fat-free mass augmentation [22]. Additional mechanistic research is highly warranted to explore what underpins the possible benefits of dietary creatine for growth and development. Establishing a threshold of dietary creatine linked with advanced growth in the youth population remains complex. We found that children and adolescents who consume extra creatine are more likely to have tall stature, with those individuals who took 0.84 to 1.49 g of creatine per day (3rd quartile) more often having tall stature than other subgroups. Nevertheless, the proportion of tall stature among participants from the highest quartile (4th) did not differ from the lowest (1st) and lower (2nd) quartiles. This perhaps suggests that consuming additional dietary creatine (>1.5 g/day) was not necessarily accompanied by a higher prevalence of tall stature and advanced growth. The daily amount of creatine consumed by children and adolescents in the 3rd quartile (mean 1.13 g) might be appropriate to facilitate growth, keeping in mind the fact that the uptake of creatine from the diet of about 1 g per day is required to achieve steady state in the adult population [4], with youth perhaps needing more dietary creatine to sustain growth and maturation.

Since meat-based foods are the primary source of creatine [10], our findings may have implications for children and adolescents who limit the intake of those foods in their diet. Previous studies reported a higher risk of growth retardation among young children with low regular meat intake, while meat consumption was associated with a reduced likelihood of stunting [23,24]. In light of our findings, the protective effect of meat consumption against stunting should emphasize the possible role of creatine, along with other food components abundant in meat, to improve nutritional practices in the pediatric population. With this in mind, creatine intake might be recognized as another nutritional factor that positively affects growth and well-being in infants and children [10]. Addressing optimal creatine consumption could be thus considered among public measures for pediatric nutrition, either via fostering diets rich in creatine-containing foods, creatine supplementation, and/or food fortification with creatine [25]. The reference intervals of dietary creatine requirements for children and adolescents remain to be elucidated.

Study strengths include using a relatively large NHANES sample, complemented with growth indicators (e.g., stature-for-age and BMI-for-age) calculated and labeled for each participant, while the correlation between creatine intake and growth indicators controlled for main demographic and nutritional variables, including gender and total protein intake. Nevertheless, several limitations have to be considered when the study results are interpreted. The cross-sectional design of the current study prevents any conclusions about a cause and effect between creatine intake and body measures and analyzing temporal changes in those variables occurring with growth. The mean dietary creatine intake has been calculated using single-day self- or proxy-reported 24-h interviews, which could be susceptible to recall bias and cannot account for a day-to-day variation. The creatine calculation method used here omitted to consider variability in creatine content across various meat-based foods and non-meat sources; the amount of creatine may differ within animal protein subgroups [11]. In addition, NHANES data provide no measure or estimation of endogenous creatine production, a possible modifying variable that could account for a total daily creatine load (a sum of creatine synthesized de novo along with creatine consumed from a diet). Finally, nutritional conditions evaluated in this cohort (NHANES 2001–2002) might be very different from the contemporary diet, and future trials should evaluate possible time-related changes in dietary creatine intake among present-day children and adolescents, also across various countries.

## 5. Conclusions

Our study demonstrated that the daily intake of creatine from a regular diet in taller children and adolescents was higher than those in shorter peers aged 2–19 years. A positive association between creatine consumption and growth remained robust after adjusting for main demographic and nutritional variables. Therefore, taking enough creatine with regular food should be considered to ensure advanced growth in U.S. children and adolescents. Future research has to monitor temporal changes in growth and dietary creatine using objective biomarkers of creatine consumption and validate our findings in interventional studies across pediatric populations.

## Figures and Tables

**Table 1:** Sample demographic and nutritional characteristics. BMI—body mass index.

Variable	
Participants, *n*	4291
Gender (%)	
Female	50.3
Age (years), mean ± SD	11.1 ± 5.3
Generation (%)	
Preschooler (2–5 years)	20.0
School-aged children (6–12 years)	33.6
Adolescent (aged 13–19)	46.4
Ethnicity (%)	
Non-Hispanic White	29.5
Non-Hispanic Black	4.7
Mexican American	30.9
Other Race	30.6
Other Hispanic	4.3
Annual household income (%)	
Less than $24,999	35.2
$25,000–$54,999	31.5
$55,000–$74,999	11.9
$75,000 and over	21.0
Body measures, mean ± SD	
Weight (kg)	48.1 ± 24.9
Height (cm)	144.7 ± 27.2
BMI (kg/m^2^)	21.0 ± 5.8
Overweight (%)	32.7
Dietary intake	
Energy (kcal) mean ± SD	2104 ± 965
Protein (g) mean ± SD	103.8 ± 65.1

**Table 2:** Body measures across creatine quartiles.

Variable	Q1	Q2	Q3	Q4	*p*	Post-Hoc *
Height (cm)	140.7 ± 28.0	137.2 ± 27.9	145.4 ± 26.5	155.3 ± 22.4	<0.001	^a b c d e f^
Weight (kg)	44.7 ± 24.5	42.1 ± 23.8	49.0 ± 25.6	56.1 ± 23.4	<0.001	^b c d e f^
BMI (kg/m^2^)	20.4 ± 5.5	20.1 ± 5.5	21.2 ± 6.0	22.1 ± 5.8	<0.001	^b c d e f^

Abbreviations: BMI—body mass index. * Superscript letters indicate a significant difference at *p* < 0.05 between individual sample pairs after post-hoc analysis, as follows: ^a^ Q1 vs. Q2, ^b^ Q1 vs. Q3, ^c^ Q1 vs. Q4, ^d^ Q2 vs. Q3, ^e^ Q2 vs. Q4, ^f^ Q3 vs. Q4.

**Table 3:** Simple and adjusted multiple regression results of the relationship between dietary creatine intake and growth indicators. BMI—body mass index.

	Crude Model			Adjusted Model		
	*B*	SE	*p*	*B*	SE	*p*
Height	0.24	0.57	<0.001	0.117	0.44	<0.001
Weight	0.20	0.35	<0.001	0.131	0.41	<0.001
BMI	0.12	0.08	<0.001	0.115	0.10	<0.001
Height-for-age	0.01	0.01	<0.001	−0.013	0.01	0.460
BMI-for-age	0.02	0.01	0.227	0.053	0.014	0.004

## Data Availability

Data described in the manuscript will be made available upon request.
